# Congenital Pyloric Atresia and Associated Anomalies: A Case Series

**Published:** 2013-10-01

**Authors:** Rahul Gupta, Varsha Soni, Praveen Mathur, Ram Babu Goyal

**Affiliations:** Department of Paediatric Surgery, SPMCHI, SMS Medical College, Jaipur, India

**Keywords:** Pyloric atresia, Epidermolysis bullosa, pyloric diaphragm, Heineke–Mickulicz pyloroplasty

## Abstract

Congenital pyloric atresia (CPA) is a very rare surgical condition. Eleven patients with the diagnosis of CPA treated at our hospital were retrospectively studied for the age at diagnosis, sex, presenting symptoms, associated anomalies, operative findings, treatment and outcome. Male: Female is 8:3. The age at diagnosis ranged from one day to three years. Associated anomalies were seen in four (36.6%). These included epidermolysis bullosa (EB) in two, oesophageal atresia with distal trachea-oesophageal fistula in one, colonic atresia in one, sensorineural deafness and dysplastic kidney in one patient. All three types of CPA were observed; six (54.5%) had type 1, four (36.3%) had type 2 and one (9%) had type 3 [(core)]. Different procedures performed were Heineke–Mickulicz pyloroplasty, Finney's pyloroplasty and gastro-duodenostomy. Post-operatively, nine out of eleven did well while other two died giving an overall survival of 81.8%. Sepsis was the cause of death in both of them.

## INTRODUCTION

Congenital pyloric atresia (CPA) is a very rare condition with an incidence of approximately 1 in 100,000 newborns and constitutes about 1% of all intestinal atresias [1]. CPA is thought to result from developmental arrest between the 5th and 12th weeks of intrauterine life [2]. Calder reported the first case of CPA in 1749 and Touroff performed the first successful operation in 1940 [3]. Commonly, CPA occurs as an isolated lesion, which has good prognosis, but it can also be seen in association with other malformations, which can have a bad impact on the ultimate outcome [4]. We report 11 cases of CPA, which is the largest series from India hitherto, [with] along with the associated anomalies, particularly the epidermolysis bullosa and sensori-neural deafness and their impact on the survival

## MATERIALS AND METHODS

We present a retrospective study of eleven cases with the diagnosis of CPA treated at our hospital between 2006 and 2012.They were studied for the age at diagnosis, sex, presenting symptoms, associated anomalies, operative findings, treatment and outcome. The details are shown in Table 1.

**Figure:**
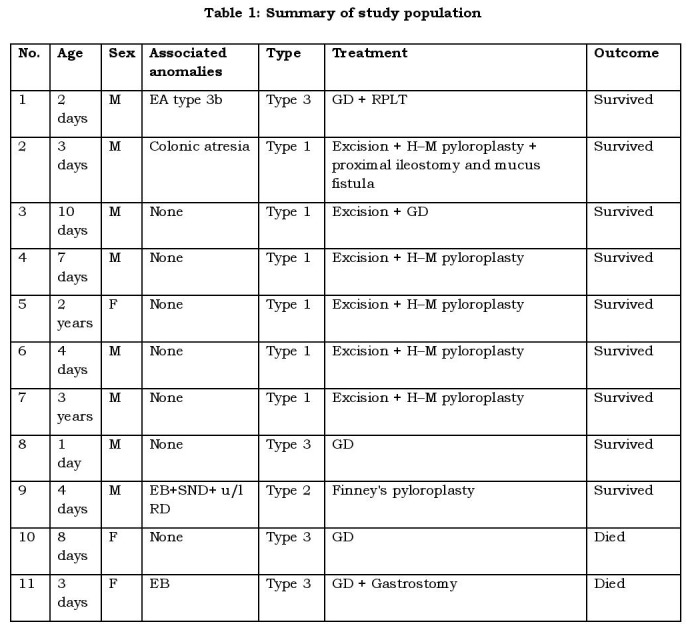
Table 1

## RESULTS

Eleven cases with the diagnosis of CPA were treated at our hospital. There were eight males and three females. The age at diagnosis ranged from one day to three years. The main presenting symptoms were non-bilious vomiting and upper abdominal distension. The two toddlers presenting late also had failure to thrive in addition to the above symptoms. One of our patients was admitted as a case of esophageal atresia with distal trachea-esophageal fistula was subsequently found to have CPA after a babygram. In nine patients, the diagnosis was made preoperatively on plain abdominal X-ray, which showed a single, large air bubble representing the dilated stomach with no gas distally. Contrast study of abdomen revealed distended stomach and non-passage of contrast beyond pylorus. The two patients presenting late were diagnosed intra-operatively. Associated colonic atresia in one patient was also diagnosed intra-operatively. [The two patients presenting late were diagnosed intra-operatively along with the one having associated colonic atresia.]

 Associated anomalies were seen in four (36.6%). These included epidermolysis bullosa (EB) in two, esophageal atresia with distal trachea-esophageal fistula in one, colonic atresia in one, sensori-neural deafness and [multicystic] dysplastic kidney[ (MCKD)] in one patient.

 All patients were operated. Six patients had pyloric diaphragms (type I). Two patients in this group presented late in toddler age group with failure to thrive, had hugely dilated stomach with small central perforation in the diaphragm. Four had pyloric atresia with a gap between the two ends (type 3) (Fig. 1). One had pyloric atresia without gap (type 2). The patients with pyloric diaphragms had excision of diaphragms with Heineke–Mickulicz pyloroplasty in five, while one had gastro-duodenostomy. Patients with pyloric atresia with a gap had gastro-duodenostomy. The patient with pyloric atresia with a gap with EB had gastrostomy and gastro-duodenostomy. Finney's pyloroplasty was performed in pyloric atresia without gap. Right posterolateral extrapleural thoracotomy with ligation of fistula and primary end to end esophageal anastomosis was performed in one patient with esophageal atresia with distal trachea-esophageal fistula, while patient with associated colonic atresia was subjected to proximal ileostomy along with distal mucus fistula.

**Figure:**
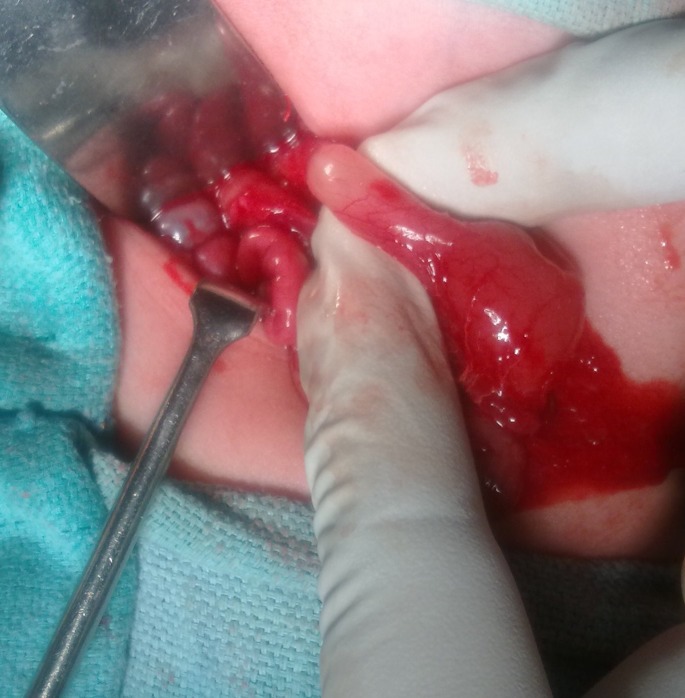
Figure 1: Operative photograph showing type- 3 CPA.

Post-operatively, nine out of eleven did well giving an overall survival of 81.8%. Out of the nine, one patient with EB with dysplastic kidney [MCKD] developed postoperative leak and was subsequently re-explored and gastrojejunostomy was performed. Two patients died in our study, one because of postoperative leak, the other due to complications of EB. Sepsis was the cause of death in both of them. 

## DISCUSSION

CPA is divided anatomically into three types [4].

Type 1 – Pyloric membrane or web (57%). 

Type 2 – Pyloric tissue replaced by solid cord /tissue (34%). 

Type 3 – Pyloric atresia with a gap between stomach and duodenum (9%). 

In our series, type 1 was the commonest (54.5%), seen in six, type 2 in one (36.3%) and type 3(9%) in four patients. Two of our patients presenting in the toddler age group had Type 1 CPA with central perforations in the diaphragms. Non-bilious vomiting are fairly constant features in CPA. Neonates with PA frequently experience apnoea, aspiration or respiratory distress due to their copious secretions. Early oro-gastric tube insertion and aspiration probably reduced the risk of early respiratory complications in these children. The diagnosis of CPA is based on the presence of a single large gastric air bubble with no gas distally on plain abdominal X-ray. Distal gas may point towards perforations in the diaphragms. Associated anomalies like junctional epidermolysis bullosa (JEB), ureteral and renal anomalies (dysplastic/ multicystic kidney, hydroureteronephrosis, ureterocele, duplicated renal collecting system, and absent bladder), aplasia cutis congenita, milia, nail dystrophy, and multiple colonic atresias have been reported and are present in 40–55% of the cases [4-6]. In our series, associated anomalies were present in 36.6% of the patients and epidermolysis bullosa (EB) was the commonest (18.2%). Intra-operatively, it is necessary to check the patency of the remaining intestines using a catheter which is passed distally and saline injected to exclude associated distal intestinal atresias which was seen in one of our patient and also to rule out Hereditary multiple intestinal atresias of the gastrointestinal tract [7]. Associated esophageal atresia with distal trachea-esophageal fistula was seen in one of our patients, it is explained by the occasional occurrence of atresias at other parts of the gastrointestinal tract [8]. The presence of associated pure esophageal atresia makes preoperative diagnosis of associated CPA impossible and it is important to exclude this at the time of gastrostomy [5].

The treatment of CPA depends on the type of atresia. Pyloric diaphragms are treated by excision and Heineke–Mikulicz pyloroplasty (involves incising the canal longitudinally, excising membrane, and transverse closure of pylorus). CPA without a gap are also treated by excision and pyloroplasty, we performed Finney's pyloroplasty in our patient and he is doing well. For pyloric atresia with a gap, the treatment is gastro-duodenostomy. Gastrostomy though not recommended as part of the operative treatment, was done in one of our patients because of associated EB. This patient latter died of complications of EB.

Isolated CPA has good prognosis. The overall high mortality exceeding 50% is attributed to the high incidence of severe and often fatal associated anomalies [4, 5]. An overall survival of 81.8% seen in our series could be explained by the fact the associated anomalies were seen in only four (36.6%) cases, while seven (63.4%) cases were without associated anomalies; it is an established fact that mortality is directly related to associated anomalies. The cause of death in the majority of the cases was sepsis and this was the case in our patients too. 

The association of epidermolysis bullosa (EB) and congenital pyloric atresia (CPA) is now known as Carmi Syndrome [9, 10]. It is rare syndrome and it has a genetic basis [11]. Epidermal bullosa (EB) is a group of hereditary disorders of skin with characteristic appearance of blistering vesicular lesions at or shortly after birth targeted to areas of minor trauma. It is categorized as EB simplex (EBS), JEB, and dystrophic EB (DEB). EB has sub-epidermal separation through the lamina lucida in the basal cell layer above and the lamina densa (basement membrane) below is diagnostic of JEB. EB is a mechano-bullous disorder and is generally a fatal disease. The incidence of EB alone is 1/300,000 and mortality with this combination is high, however, healthy long-term survival is documented in the literature [12]. There are no definite treatment modalities in the treatment of EB. The treatment includes appropriate dressing, infection control and nutritional supplements. Topical steroids may be used for the management of local inflammation.

One of our CPA patients who had an associated EB, sensori-neural deafness and dysplastic kidney [MCKD] survived and is on regular follow up. The cause of deafness could be due the similar ectodermal origin of inner hair cells and the skin. The diagnosis of EB requires electron microscopic studies and same is not available in our center.

The other EB patient who was last case in our study had multiple, fluid-filled, bullous lesions of the skin and mucous membranes, subsequently had peeling of skin on lower thigh, leg and dorsum of foot and hand and parietal eminences of the scalp (Fig.2). Severe hypoproteinemia, hyponatremia, anemia, septicemia followed and patient died. The association of CPA with EB should not preclude surgical treatment as per the author and non-operative management of PA is inappropriate in the stable neonate with EB [13].

## Footnotes

**Source of Support:** Nil

**Conflict of Interest:** None

